# Impact of the acquired subgenome on the transcriptional landscape in *Brettanomyces bruxellensis* allopolyploids

**DOI:** 10.1093/g3journal/jkad115

**Published:** 2023-05-24

**Authors:** Arthur Jallet, Anne Friedrich, Joseph Schacherer

**Affiliations:** CNRS, GMGM UMR 7156, Université de Strasbourg, 67000 Strasbourg, France; CNRS, GMGM UMR 7156, Université de Strasbourg, 67000 Strasbourg, France; CNRS, GMGM UMR 7156, Université de Strasbourg, 67000 Strasbourg, France; Institut Universitaire de France (IUF), 75005 Paris, France

**Keywords:** polyploids, subgenome, transcriptional landscape, yeast, *Brettanomyces bruxellensis*

## Abstract

Gene expression variation can provide an overview of the changes in regulatory networks that underlie phenotypic diversity. Certain evolutionary trajectories such as polyploidization events can have an impact on the transcriptional landscape. Interestingly, the evolution of the yeast species *Brettanomyces bruxellensis* has been punctuated by diverse allopolyploidization events leading to the coexistence of a primary diploid genome associated with various haploid acquired genomes. To assess the impact of these events on gene expression, we generated and compared the transcriptomes of a set of 87 *B. bruxellensis* isolates, selected as being representative of the genomic diversity of this species. Our analysis revealed that acquired subgenomes strongly impact the transcriptional patterns and allow discrimination of allopolyploid populations. In addition, clear transcriptional signatures related to specific populations have been revealed. The transcriptional variations observed are related to some specific biological processes such as transmembrane transport and amino acids metabolism. Moreover, we also found that the acquired subgenome causes the overexpression of some genes involved in the production of flavor-impacting secondary metabolites, especially in isolates of the beer population.

## Introduction

Genetic variants shape the phenotypic diversity observed between individuals of the same species. These variants also affect gene expression levels, and divergence at the transcriptional level undoubtedly represents a key driver for phenotypic diversity as well as evolution. In addition, gene expression levels can be considered as molecular traits that might bridge the gap between genotypes and phenotypes (Gibson and Weir 2005; Rockman and Kruglyak 2006; Albert and Kruglyak 2015). Surveys exploring the natural variation in gene expression between individuals from different populations have revealed that a significant proportion of the transcriptome evolves in a way consistent with neutrality, with few exceptions (Oleksiak *et al*. 2002; Whitehead and Crawford 2006). Several frameworks that contrast observed variation in gene expression between groups of individuals with different evolutionary patterns are now available to distinguish signatures of positive selection from purifying or neutral selection (Fay and Wittkopp 2008; Romero *et al*. 2012; Hodgins-Davis *et al*. 2019; Price *et al*. 2022). However, there is currently a lack of large-scale studies that explore natural variation in gene expression and its adaptive potential. In fact, most examples of putative adaptive effects come primarily from studies analyzing the progeny of a single cross (Salinas *et al*. 2016), groups with few individuals (Ibáñez *et al*. 2017; Jenull *et al*. 2021), as well as emphasizing tolerance to specific perturbations such as mutations (Vu *et al*. 2015) or various stresses (Fay *et al*. 2004; Kvitek *et al*. 2008; Ibáñez *et al*. 2017).

In addition, several haplotypes coexist within diploid or polyploid organisms, and the expression level may differ between them. This differential expression between the two parental alleles is called allele-specific expression (ASE) (Pastinen 2010; Cleary and Seoighe 2021), and the fraction of a diploid genome affected by ASE has been shown to affect a non-negligible fraction of genes in several organisms (Zhang and Borevitz 2009; Muzzey *et al*. 2014; The GTEx Consortium 2017; Hoguin *et al*. 2021). As shown in the yeast *Saccharomyces cerevisiae*, both *cis*- and *trans*-effects can explain ASE (Emerson *et al*. 2010; Salinas *et al*. 2016; Villarroel *et al*. 2021). Regarding variation in expression of more distant coexisting genomic copies, analyses of artificial crosses between species (Tirosh *et al*. 2009; Lairón-Peris *et al*. 2020), natural polyploid species (Zhao *et al*. 2020; Timouma *et al*. 2021), and recently introgressed genomic regions indicate that expression of certain genes could sometimes be biased in favor of one of the parental species (Brion *et al*. 2015).

To have a deeper insight into the differential expression of coexisting genomes and their consequences, we sought to explore and compare the transcriptomes of a large set of *Brettanomyces bruxellensis* yeast isolates. *Brettanomyces bruxellensis* is exclusively found associated with anthropized fermented substrates (Smith and Divol 2016). While being one of the major spoilers in wine, it can also have a beneficial impact on other fermented beverages such as some specific beers and kombucha (Harrouard *et al*. 2023, Schifferdecker *et al*. 2014). *Brettanomyces bruxellensis* is composed of isolates exhibiting different genomic architectures with various ploidy levels and characterized by a complex population structure with several well-separated anthropized populations (Avramova, Cibrario, *et al*. 2018; Gounot *et al*. 2020). A recent study confirmed the existence of six well-resolved populations structured by the ecological origins of the isolates and highlighted a high genomic content variation between these populations (Eberlein *et al* 2021). It also showed that the ploidy is conserved within the populations and, more importantly, that three populations, namely Teq/EtOH, Beer and Wine 1, acquired an additional highly divergent haploid genome through three independent interspecific hybridization events. These three populations are therefore composed of allotriploid individuals whose genomes consist of a primary diploid (2n) genome common to all isolates of the species and an extra haploid (1n) subgenome specific to each of them. Of these populations, the Teq/EtOH population, which includes isolates from tequila and bioethanol production plants, was found to be the most genetically diverse, consistent with it being the oldest.

The coexistence of these genomes of different origins in several populations of *B. bruxellensis* is particularly interesting and raises questions about the behavior of these coexisting genomes at the transcriptional level. To date, data are sparse when it comes to studying gene expression and its differential regulation between *B. bruxellensis* populations, as only very few isolates have been explored, preventing a full view of the transcriptional variation (Piškur *et al*. 2012; Tiukova *et al*. 2013; Godoy *et al*. 2016; Varela *et al*. 2019; Valdetara *et al*. 2020; He *et al*. 2022).

Here, we generated and compared the transcriptomes of a set of 87 *B. bruxellensis* isolates, selected as being representative of the genomic diversity of this species. Our analysis revealed that the additional subgenomes have a significant impact on the transcriptional patterns and allowed discrimination of allopolyploid populations. In addition, differential expression patterns reveal clear transcriptional signatures related to specific populations. The transcriptional variations observed are related to some specific biological processes such as transmembrane transport, amino acids metabolism, and secondary metabolites.

## Materials and methods

### Growth culture and mRNA extractions

For this study, we used 87 *B. bruxellensis* strains selected from a collection of one thousand (Supplementary Table 1). Among them, 45 are from one of the three allotriploid (2n + 1n) populations: 9 come from the Teq/EtOH population, 30 from the Beer population, and 6 from the Wine 1 population. This proportion of allopolyploid isolates in our data set reflects the proportion found in the whole collection (Harrouard *et al*. 2023). Non-allopolyploid isolates included in the present study are distributed as follows: 9 from the autotriploid (3n) Wine 2 population, 26 from the diploid Wine 3 population, and 7 from the diploid Kombucha population.

Liquid glycerol stocks were used to inoculate precultures in yeast extract peptone dextrose (YPD) for 2 days in 96 wells deep-well plates. Precultures were then diluted to optical density (OD) = 0.08 (Tecan Infinite F200 Pro micro-plate spectrophotometer) in 96 wells deep-well plates containing 1 mL of fresh YPD medium and incubated at 30°C at 130 rpm until exponential growth was established. Depending on the strain, exponential growth was reached at OD = [0.5–0.6]. At this stage, cultures were transferred to 0.45 μM 96-well filter plates (Norgen, #40008) and vacuum-filtered (VWR Multi-well plate vacuum manifold, #16003-836). After filtration of the entire medium, filter plates were immediately snap-frozen in liquid nitrogen and stored at −80°C for mRNA extraction.

mRNA was extracted with the Dynabeads mRNA Direct Kit (ThermoFisher, #61012) following manufacturer's instructions, with modifications for 96-well plates as previously described (Albert *et al*. 2018). Cells were lysed in 1.1 mL plates (Axygen plates, VWR, ref. 736-0339) using the provided lysis buffer, glass beads (Sigma Aldrich, #G8722), and a bead-beater (VWR STAR-BEATER, #412-0167). Magnetic beads covered with poly(dT) oligonucleotides were used for mRNA isolation in PCR plates. A volume of 11 μL of purified mRNA was finally eluted, and 10 μL was used for library preparation.

### Library preparation and sequencing

cDNA libraries were prepared using the NEBNext Ultra II Directional RNA Library Prep Kit for Illumina (NEB, #E7765). The protocol applicable for purified mRNA was performed according to the manufacturer's instructions. Briefly, mRNA was heated for 15 minutes at 94°C before conducting successively the first and second cDNA strand synthesis reactions. The cDNA was purified with magnetic beads and eluted in Tris-EDTA (TE). NEBNext adaptors were ligated to cDNA using the provided mix and buffer, the USER enzyme was activated at 37°C for 15 minutes to release single-stranded and adaptor-ligated cDNA molecules, and the product of the ligation reaction was purified using magnetic beads and eluted in TE. The last step was a 9-cycle PCR-amplification of the library, using Illumina dual barcode indexing (i5 and i7) for multiplexing. For quality check, an equimolar mix of all samples was prepared, and the integrity of the pool was checked by migration on a 1% gel agarose. Samples were individually quantified using the Qubit dsDNA HS Assay Kit (Invitrogen). The final pool of cDNA was sequenced on an Illumina NextSeq sequencer at the EMBL Genomics Core Facilities, Heidelberg, Germany, using 75 bp single-end sequencing.

### Polishing of haploid extra subgenome assemblies

In the context of a previous study (Eberlein *et al*. 2021), one representative assembly of the haploid acquired subgenome was constructed for each allotriploid population, namely Teq/EtOH, Beer and Wine 1 (Supplementary Table 2). The strains chosen as reference for these populations were YJS7890, YJS7895, and YJS8039, respectively. In order to refine these de novo long reads assemblies, the Illumina reads corresponding to these haploid subgenomes were identified through competitive mapping between the diploid reference sequence (YJS5431, Fournier *et al*. 2017) and the respective haploid assemblies, using BWA (Li and Durbin 2009) v0.7.17 with the default settings (mem algorithm). The haploid-specific long reads were retrieved through competitive mapping with Minimap2 (Li 2018) – parameter -ax map-ont.

These haploid-specific sets of reads were then used for the respective haploid assemblies polishing through three rounds of Racon (Vaser *et al*. 2017), with default parameters, followed by two rounds of Medaka (medaka: Sequence correction provided by ONT Research 2018, https://github.com/nanoporetech/medaka), with -m set to r941_min_high_g303 and finally three rounds of Pilon (Walker *et al*. 2014), with default parameters.

### Orthologous gene transfer from the reference genome to haploid assemblies

To localize the orthologous genes within the acquired subgenomes, we used a simple scheme enabling gene transfer from the diploid reference genome (Fournier *et al*. 2017). Features annotated as protein-coding genes or pseudogenes in the reference (5,206 in total) were blasted as queries onto the haploid subgenomes refined assemblies. The following blast parameters were used: -outfmt 7 -max_target_seqs 3 -evalue 1e-10 -perc_identity 70. We increased the stringency of the blast by keeping only targets that were at least identical to the query at 85% and that were above 95% of the query length. Overall, 4,572 genes were recovered in the Teq/EtOH haploid subgenome, 3,622 in the Beer one, and 3,930 in the Wine 1 one.

### Functional annotation through gene ontology mapping

We noticed that among the 5,206 genes annotated on the reference diploid genome, 2,375 have associated orthologs, mostly from *S. cerevisiae*. Gene ontology (GO) terms associated with these genes were retrieved from the Uniprot database (https://www.uniprot.org/id-mapping). Among them, 64 lack a GO cellular component (CC) annotation, 139 lack a GO biological process (BP) annotation, and 275 lack a GO molecular function (MF) annotation. Functional annotations were then transferred to orthologs detected in the different haploid subgenomes.

### Mapping of RNAseq reads for allopolyploid and non-allopolyploid samples

To map our RNAseq reads, we used two distinct strategies depending on whether samples were from allopolyploid individuals (i.e. from Teq/EtOH, Beer, and Wine 1 populations) or non-allopolyploid individuals (i.e. from the Wine 2, Wine 3, and Kombucha populations). For non-allopolyploid samples, reads were mapped to the *B. bruxellensis* reference genome, which comes from a diploid strain isolated in an Italian winery. For allopolyploid (2n + 1n) samples, we used a competitive mapping approach, using concatenated genomes to map our reads.

For each allotriploid group, we concatenated the reference diploid genome to the respective acquired haploid subgenome assembly. Reads were mapped to their respective concatenated genomes, each consisting of a diploid part and a haploid part. This competitive mapping approach allows reads to map to the subgenomes they most probably come from. Reads were aligned with STAR (Dobin *et al*. 2013), using the following parameters: –outFilterMismatchNmax 4, –outSAMmultNmax 10, –outSAMtype SAM, –outFilterType BySJout, –alignIntronMin 20, –alignIntronMax 200, –alignSJoverhangMin 6. Multi-mapped reads were handled with the rescue method implemented in Cufflinks (Trapnell *et al*. 2013), with the -u option. This allowed multi-mapped reads to be assigned to one specific location based on the coverage of uniquely mapped reads surrounding the different multi-mapped locations.

For the minimum evolution tree construction, the mapping of the reads was also performed with STAR, applying the same parameters, but solely on the reference diploid genome. SNPs were called by following GATK recommendations (https://github.com/gatk-workflows/gatk3-4-rnaseq-germline-snps-indels), and a gvcf matrix was constructed with the GenotypeGVCFs options. The 345,272 biallelic segregating sites were used to construct a minimum evolution tree with the R packages ape and SNPrelate.

### Gene expression quantification relatively to haploid library for comparison across allopolyploid samples

When comparing the expression profiles related to one of the subgenomes across our allopolyploid populations, we took into account the fact that within each library, only a fraction of reads was assigned to each of these subgenomes. To be able to compare samples regarding their haploid or diploid expression, reads restricted to either the primary or the acquired subgenome were retrieved and normalized according to the total number of reads that specifically mapped on these subgenomes.

### Identification of differentially expressed genes

The differential expression analysis was performed with DESeq2 (Love *et al*. 2014) on read counts. Library size normalization was performed with the default method implemented in DESeq2. We identified differentially expressed genes in each population using the *nbinomWaldTest()* function, by contrasting expression in the focal population versus expression in samples from all the other populations as a whole. Genes were considered differentially expressed (DE) if their false discovery rate (FDR)-corrected *P*-values were below 0.05 and the absolute log2 fold-change above 1 (corresponding to an upregulation or downregulation by a minimum of 2-fold) (Supplementary Table 3).

### Functional enrichment analysis

Based on gene ontology information, we used the GOseq R package (Young *et al*. 2010) to detect GO BP, GO MF or GO CC terms that are enriched in our sets of differentially expressed genes. Terms were considered significant if the corrected *P*-value of their enrichment score was above 0.1 (Supplementary Table 4).

### Analysis of genes involved in glucose catabolism

We used pathways annotations embedded in the SGD database (https://pathway.yeastgenome.org/, last accessed 2023 April 24) to retrieve genes involved in glycolysis, fermentative and respiratory processes. The following four pathways were considered: “Glycolysis I (from glucose 6-phosphate)” (15 genes), “Superpathway of glucose fermentation” (15 genes non-redundant with the previous pathway), “TCA cycle, aerobic respiration” (21 genes), and “aerobic respiration, electron transport chain” (27 genes). For each pathway, 13, 9, 18, and 15 *B. bruxellensis* orthologs were found, respectively.

### Identification of population specific transcriptomic signatures

We defined a gene as being a transcriptomic signature in one population if it was strongly over- or under-expressed in this group. To detect transcriptomic signatures, we applied stringent criteria on our sets of differentially expressed genes. The absolute log2 fold-change of expression in the considered population compared to the rest had to be above 1.6 (corresponding to an upregulation or downregulation by a minimum of 3-fold), and the adjusted *P*-value had to be among the top-half of the *P*-values of genes that are DE in the same direction (i.e. either upregulated or downregulated). With this definition, we identified 54 signatures in Teq/EtOH, 58 signatures in Beer, 77 signatures in Wine 1, 9 signatures in Wine 2, 73 signatures in Wine 3, and 26 signatures in Kombucha (Supplementary Table 5).

### Predictions to infer genes departing from the sample-specific 2n versus 1n relationship

For each allotriploid sample, we performed a linear regression between counts from the primary (2n) and acquired (1n) subgenomes, using orthologs present in both subgenomes. Instead of defining a hard threshold to detect genes expressed at different levels in the two subgenomes, we used the R *predict()* function to evaluate, within each sample, which genes deviated significantly from the prediction based on the regression. Genes detected by this way are those that do not follow the overall trend between the 2n versus 1n relationship in the considered sample. This approach allows to account for the fact that samples display 2n versus 1n relationships that can vary significantly.

### Definition of genes with consistent over- or under-expression in one subgenome of allopolyploid populations

Based on the number of samples in which a gene deviated from the 2n versus 1n relationship, we defined genes that consistently fell out of predictions in most samples. To be considered consistently expressed at different levels between the two subgenomes of a given population, a gene had to deviate in at least 25 out of the 30 Beer samples, in at least 7 out of the 9 Teq/EtOH samples, or in at least 5 out of the 6 Wine 1 samples. To visualize expression differences of individual genes between the two subgenomes across multiple samples, we corrected counts by library size of each subgenome, using the sum of counts of orthologs present in both subgenomes. This visualization fits with the rationale used to detect outliers within each sample, which is based on the deviation from sample-specific 2n versus 1n relationship. Paired t-tests were performed to compare gene expression from the two subgenomes, and *P*-values for each population were corrected for multiple comparisons using the Holm method.

## Results

**Fig. 1.**
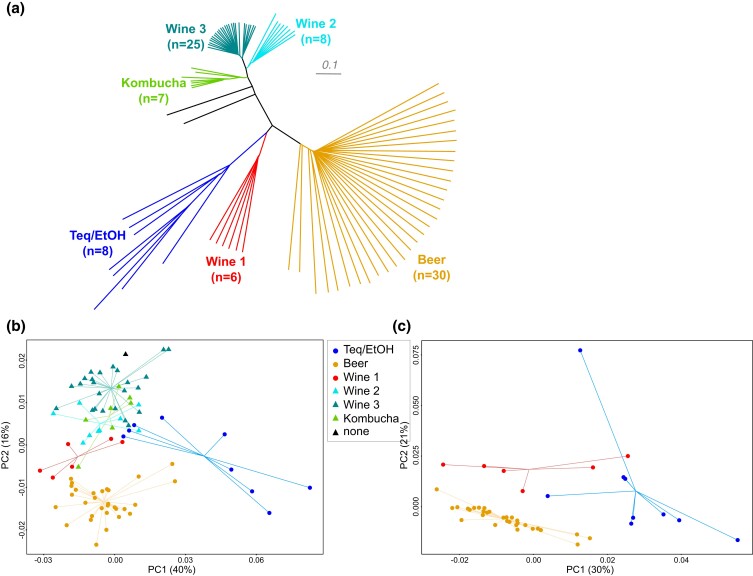
Intra- and inter-population variations of expression profiles of the primary diploid and acquired haploid subgenomes. a) Minimum evolution tree constructed on the basis of 345,272 biallelic single nucleotide variants detected through the mapping of RNAseq reads on the primary diploid subgenome. b) Principal component analysis from all reads that mapped to the primary diploid subgenome, which is present in all isolates of the *Brettanomyces bruxellensis* species. Read counts were normalized by accounting for the fraction of reads assigned to the diploid subgenome during competitive mapping and log2 transformed. c) PCA based on reads that mapped to the acquired haploid subgenomes of allotriploid isolates (Teq/EtOH, Beer, and Wine 1) during competitive mapping. Genes shared by the three acquired subgenomes of these populations were used, and counts were normalized by the total number of reads assigned to these 2,819 genes, before log2 transformation. For both PCA, symbol shapes represent allotriploid (filled circles) and non-allotriploid (triangles) populations and samples are linked to the centroid of the population they belong to.

To study gene expression variation within the *B. bruxellensis* species, we selected a set of 87 isolates representative of the six previously identified populations, namely the Beer, Kombucha, Teq/EtOH, Wine 1, Wine 2, and Wine 3 clades (Avramova, Cibrario, *et al*. 2018) (Fig. 1a). These populations are characterized by their main substrate of isolation as well as the ploidy of related isolates, which varies from diploid to tetraploid. Long-read sequencing of 71 isolates recently confirmed that ploidy is well conserved within each population and further refined the genomic architecture of triploid populations (Eberlein *et al*. 2021). Our previous study clearly highlighted the different trajectories of polyploidization leading to auto- as well as allopolyploids. Whereas the Wine 2 population is composed of autotriploids (3n), the Wine 1, Teq/EtOH, and Beer populations are allotriploids (2n + 1n). These latter are composed of a diploid primary genome, present in all isolates of the species *B. bruxellensis*, as well as an additional haploid subgenome resulting from hybridization events. These subgenomes are collinear to the primary genome but very divergent, with a genetic divergence greater than 2.5%. In addition, the acquired haploid copies are different depending on the population, highlighting clade-specific allopolyploidy events (Eberlein *et al*. 2021). Here, we generated the transcriptomes of 33 diploid, 9 autotriploid (3n), and 45 allotriploid (2n + 1n) isolates (Supplementary Table 1), with most of them (61/87) belonging to the set of the 71 previously characterized isolates.

### Annotation and gene content assignment to the different acquired subgenomes

In total, three different allopolyploidization events related to the Wine 1, Teq/EtOH, and Beer populations were detected in the *B. bruxellensis* species. For each of these events, the acquired haploid subgenome is different and therefore a different haploid subgenome was chosen as a reference for the Wine 1, Teq/EtOH, and Beer clades. Representative assemblies of these previously generated subgenomes were refined by short and long reads polishing steps (Supplementary Table 2). These subgenomes are significantly shorter (from 9.2 Mb to 11.3 Mb for the Beer and Teq/EtOH, respectively) compared to the diploid primary genome (13 Mb). This reduced size can be attributed to the reorganization of the polyploid genome through large loss of heterozygosity (LOH) events. Interestingly, it was shown that these LOH regions display a well conserved pattern within populations, with only a few and small LOH events private to specific strains (Eberlein *et al*. 2021).

The annotation of the reference diploid genome was used to carry out an annotation transfer on these assemblies by similarity searches (Gounot *et al*. 2020). Among the 5,206 protein-coding genes present in the reference primary genome, we found 4,572 (88%), 3,622 (70%), and 3,930 (75%) genes in the Teq/EtOH, Beer, and Wine 1 acquired subgenomes, respectively. In addition, a total of 2,819 orthologous genes were shared by these three acquired subgenomes (Supplementary Fig. 1). The fraction of protein-coding genes that were transferred onto each acquired haploid genome assembly is consistent with the map of LOH regions (Supplementary Fig. 1).

### Subgenome expression profiles reveal the population structure

To assess gene expression variation in *B. bruxellensis*, we sequenced the total mRNA from the previously described set of 87 isolates during exponential growth in complete medium (Supplementary Table 1). Due to the variation in the genomic architecture and more specifically the presence of various subgenomes in allotriploids, the transcriptomic analyses were carried out with different reference genomes. For the diploid and autopolyploid isolates (i.e. Wine 2, Wine 3, and Kombucha), reads were directly mapped on the primary reference genome (Fournier *et al*. 2017). In contrast, reads coming from allotriploids isolates were mapped to assemblies that concatenate the primary reference diploid genome and the acquired subgenome representative of the population of interest (Wine 1, Teq/EtOH, or Beer). This strategy makes it possible to avoid mapping biases due to the high genetic divergence between the primary and the acquired subgenomes (>2.5%) in the case of the allotriploid isolates.

We first considered reads that specifically mapped to the diploid primary genome. A principal component analysis (PCA) based on these read counts reveals three main clusters (Fig. 1b). In addition to the main cluster corresponding to the Beer population, all diploid and autotriploid isolates (Wine 2, Wine 3, and Kombucha) as well as four Teq/EtOH isolates clustered together. Interestingly, all Wine 1 samples fall between these two well-defined clusters. The third cluster is further away from the others and includes five of the nine Teq/EtOH samples. This observation strongly suggests that the presence of a divergent subgenome impacts the expression of the primary genome to a great extent. However, notable differences can be seen even across transcriptomes of non-allopolyploid clades, indicating transcriptomic rewiring per se and independent of ploidy level.

We then focused on the gene expression profiles of the acquired subgenomes and subjected the expression of the 2,819 genes shared by the three subgenomes to a PCA (Fig. 1c). This analysis clearly separates the three populations. Beer samples cluster together and apart from the other populations. For the Teq/EtOH and Wine 1 populations, the samples are scattered along the range of one of the major components, i.e. PC1 for Wine 1 and PC2 for Teq/EtOH. This observation clearly shows a higher intra-population subgenome expression diversity compared to the Beer population. It should be noted that this clustering is neither a consequence of the different number of annotated genes on the three different acquired subgenomes nor of the overall proportion of reads aligned to the primary diploid versus the haploid subgenome since we normalized counts by the total number of reads, which have been assigned to the 2,819 genes commonly present in all subgenomes.

**Fig. 2.**
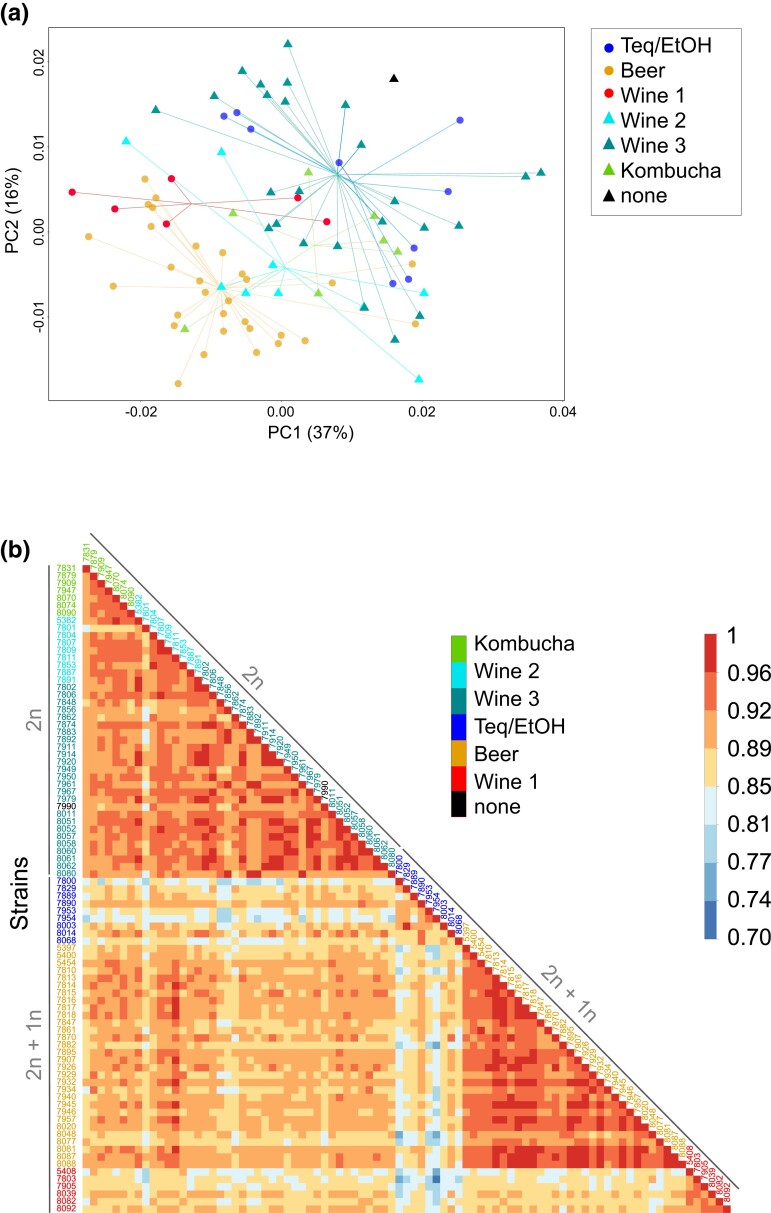
Distinctive patterns of global expression profiles across populations. a) For allotriploid samples (Teq/EtOH, Beer, and Wine 1), reads that mapped to genes present on the acquired haploid subgenome were summed with reads mapped to their orthologs of the primary genome, resulting in collapsed read counts. Collapsed counts from allotriploids and counts from non-allopolyploids were used to perform a principal component analysis after normalization by total library size and log2 transformation. Colors stand for populations, and symbol shapes represent allotriploid (filled circles) and non-allotriploid (triangles) populations; samples are linked to the centroid of the population they belong to. b) Matrix of pairwise correlation between isolates expression profiles. Correlation coefficient was computed using the Spearman correlation. Collapsed counts were used for allotriploid samples, and counts were normalized by total library size. Samples were intendedly ordered by population, as shown by the color of the isolate labels.

To obtain a more global view of gene expression variation, we summed the read counts from the primary and acquired genomes in the case of the allotriploids when orthologs exist in both of them. These collapsed counts allowed for direct comparison with the other isolates from the collection (i.e. diploid and autotriploid isolates), for which reads were only mapped to the reference primary genome. While no clear grouping pattern emerges from the PCA (Fig. 2a), a pairwise correlation matrix based on these whole-genome expression profiles allowed to distinguish three clusters (Fig. 2b). Among them, one is related to the Beer and one to the Wine 1 allopolyploid populations. The third one includes all the non-allopolyploid samples (Wine 2, Wine 3, and Kombucha), further confirming the impact of the highly divergent haploid subgenome in shaping transcriptomic landscape. Regarding the Teq/EtOH allopolyploid population, its global expression pattern diverges compared to other populations but, more importantly, profiles within the population are much more heterogeneous compared to what is observed within other clades.

Overall, based on the different PCA analyses on the primary and acquired subgenomes or both, it seems to be clear that the different polyploidization trajectories have an important impact on the transcriptional landscape within the *B. bruxellensis* species.

### Variable expression of genes involved in transport differentiates *B. bruxellensis* populations

We next focused on genes that are DE by comparing each clade to the rest of the populations, using the previously described collapsed counts. We identified 202 differentially expressed genes per population on average, ranging from 43 to 324 genes in the Wine 2 and Wine 1, respectively (Supplementary Table 3).

**Fig. 3.**
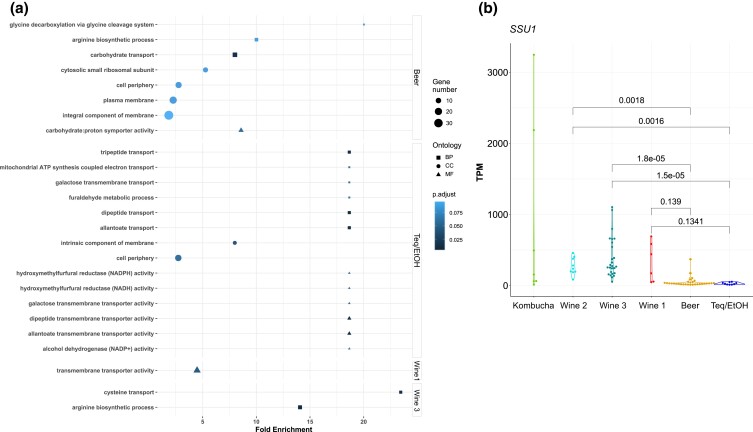
Enrichment in transmembrane transport in sets of differentially expressed genes. a) A test of functional enrichment based on gene ontology terms was performed on the sets of differentially expressed (DE) genes in each population. Ontology terms are classified in three broad categories: BP for biological process (squares), CC for cellular component (filled circles), and MF for molecular function (triangles). For each term found as significantly over-represented, dot size indicates the number of differentially expressed genes annotated with this term, and shades of blue indicate the adjusted *P*-value (Benjamini–Hochberg correction) of the enrichment. Position of dots along the *x*-axis is proportional to the enrichment score, defined as the ratio between the proportion of DE genes with the focal GO term in the set of DE genes to the proportion of genes annotated in the genome with the focal term. Only populations for which at least one functional term was found over-represented in the set of DE genes are represented. b) Collapsed expression level in transcripts per million (TPM) of the sulfite efflux pump *SSU1* (locus_tag=DEBR0S2e12442g). *P*-values indicate the FDR-corrected significance of pairwise t-tests (Welch's t-tests assuming unequal variance), and only pairwise comparisons between the three “Wine” populations and the Beer and Teq/EtOH populations are reported. We note that testing the overall expression across the three “Wine” groups against the Teq/EtOH and Beer groups pooled together results in a highly significant difference (*P* = 2.0e^−09^, Welch's t-test). When each group was contrasted to all the others, *SSU1* was downregulated in both Beer and Teq/EtOH, while it was upregulated in Wine 3 and Kombucha.

The analysis of gene ontology enrichment sheds more light on the processes that differentiate populations from each other (Fig. 3a and Supplementary Table 4). Overall, several DE sets were enriched for genes related to transmembrane transport activity, related to either carbohydrates, amino acids, or di- and tripeptides. We noticed that isolates from the Wine 1 and Wine 3 populations generally express genes related to amino acids and peptides transporters at higher levels compared to other populations, especially compared to the Beer clade. For example, the vacuolar permease of basic amino acids transporter *VBA2* and the allantoate and dipeptides plasma membrane transporter *DAL5* were upregulated in the Wine 1 population, while transporters of sulfur containing amino acids *MUP1* and *YCT1* were upregulated in the Wine 3 population. On the other hand, the general amino acid permease *GAP1* and dipeptides transporter *DAL5* were both downregulated in the Beer population. In the Wine 3 population, we also note a specific and strong enrichment for arginine biosynthesis-related processes. Finally, regarding carbohydrates, many transporters are downregulated in the Beer population (*GAL2*, *HXT7*, *MAL11*, and *STL1*), while others are upregulated in the Teq/EtOH population (*GAL2* and *MAL31*). Interestingly, the mitochondrial dicarboxylate carrier *DIC1*, which mediates the entry of malate, succinate, and oxaloacetate into the mitochondrial matrix, is upregulated both in Beer and Teq/EtOH populations.

As a Crabtree-positive yeast, *B. bruxellensis* is able to produce ethanol even under aerobic conditions in the presence of high glucose concentration. We therefore examined the expression of genes related to glucose catabolism in our set of 87 isolates, and more specifically, the genes involved in the cytosolic pathway of glucose degradation (glycolysis and fermentation) and in the subsequent mitochondrial processing of pyruvate (tricarboxylic acid cycle—TCA cycle—and electron transport chain). No clear pattern of population differentiation populations emerged (Supplementary Fig. 2), consistent with the fact that none of these pathways were found to be overrepresented in our analysis of differentially expressed genes between populations. This suggests that the balance between fermentative and respiratory processes is not a main driver of transcriptomic differences across populations in the condition studied.

Due to the widespread use of sulfur dioxide (sulfite) in the wine industry and since previous studies have reported that tolerance to this compound varies among *B. bruxellensis* populations (Avramova, Cibrario, *et al*. 2018; Avramova, Vallet-Courbin, *et al*. 2018), we finally examined the expression of the *SSU1* gene (locus_tag=DEBR0S2e12442g), encoding the sulfite efflux pump gene. This latter was significantly downregulated in Beer and Teq/EtOH populations whereas it was significantly upregulated in Wine 3 and Kombucha (Fig. 3b and Supplementary Table 3). More generally, we found that, except for the Kombucha population, samples from the non-wine groups expressed the *SSU1* gene at lower levels compared than the wine groups (*P* = 2.0e^−09^, Welch's t-test), which is consistent with results suggesting that sulfite tolerance is adaptive for some wine isolates (Avramova *et al*. 2019).

### Transcriptional signatures related to specific populations and anthropic niches

**Fig. 4.**
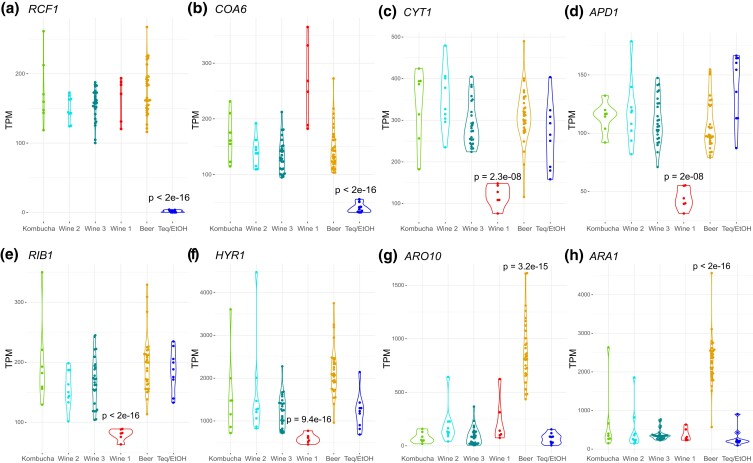
Transcriptomic signatures differentiating populations of *Brettanomyces bruxellensis*. a and b) Expression of two Teq/EtOH transcriptomic signatures across populations: the respiratory supercomplex factor (*RCF1*) and the cytochrome oxidase assembly gene (*COA6*). c to f) Expression of four Wine 1 transcriptomic signatures across populations: the cytochrome c1 gene (*CYT1*), the hydrogen peroxide resistance gene (*APD1*), the riboflavin biosynthesis gene (*RIB1*), and the glutathione peroxydase hydroperoxyde resistance gene (*HYR1*). g and h) Expression of two Beer transcriptomic signatures across populations: the decarboxylase-encoding *ARO10* gene and the oxidoreductase-encoding *ARA1* gene. For all panels, *P*-values indicate the significance of testing collapsed expression level in the clade in which the considered gene is a signature against other clades as a whole, using a t-test assuming unequal variance (Welch's t-test). For each panel, expression levels are given in transcripts per million (TPM).

In order to characterize transcriptomic signatures, we searched for genes that display strong over- or under-expression in each population compared to the others (Supplementary Table 5 and *Methods* section). Some transcriptomic signatures related to genes involved either in central metabolism, response to stressors, nutrient uptake, or production of secondary metabolites impacting flavor have been highlighted (Fig. 4). For example, two genes involved in the respiratory electron transport chain were specifically downregulated in the Teq/EtOH population: *RCF1*, encoding a subunit of the cytochrome c oxidase (complex IV), and *COA6* involved in the assembly of the cytochrome c oxidase. Another gene involved in the mitochondrial respiratory chain, *CYT1*, that encodes part of the cytochrome bc1 complex, was also detected as strongly downregulated, but this time specifically in the Wine 1 population. Three genes involved in protection against oxidative stress were also expressed at lower levels in this population: the hydrogen peroxide resistance gene *APD1*, the riboflavin biosynthesis *RIB1* gene, and the glutathione peroxidase hydroperoxide resistance gene, *HYR1*. The lowering of oxidative stress defense could be linked to a decrease in electron leakage along the mitochondrial respiratory chain, considering the key role of *CYT1* in electron transfer and its under-expression in this population. Regarding signatures involved in transport, we interestingly observed that DEBR0S2e13630g, an ortholog to the glycerol transporter *STL1*, is expressed at very low levels in all populations but the Beer population (Supplementary Fig. 3). The expression of the second paralog of this gene, namely DEBR0S2e02784g, is much higher (median of expression at 250 transcripts per million (TPM) across samples, Supplementary Fig. 3) and uniform across all populations. *STL1* is a plasma membrane proton symporter allowing active influx of glycerol into the cell, and whose expression is repressed by glucose. Glycerol is known to influence the beverage body and fullness, as well as flavor intensity (Zhao *et al*. 2015).

Finally, the *ARO10* and *ARA1* genes, both involved in aroma production, were also strongly and specifically upregulated in the Beer population while being downregulated in the Wine 3 population (Figs. 4g and h). ARO10 is a key enzyme in the Ehrlich pathway, which is involved in the synthesis of higher alcohols (also known as fusel alcohols) from branched and aromatic amino acids (Pires *et al*. 2014). Consistently, it has been shown that *ARO10* is an important gene in the production of the fusel alcohol isobutanol (Piškur and Compagno 2014), what is looked for in certain beers (Serra Colomer *et al*. 2019). Regarding ARA1, it is a dicarbonyl reductase enzyme reducing α,β dicarbonyl compounds known as vicinal diketones (VDKs). The two most prominent VDKs produced during fermentation are 2,3-pentanedione and diacetyl (2,3-butanedione), which are undesirable at moderate to high concentrations. For this reason, low levels of VDKs are usually a target in brewing. Importantly, ARA1 converts these VDKs into more neutral compounds that are less susceptible to be regarded as undesirable in brewing (Ferreira and Guido 2018). By overexpressing the *ARO10* and *ARA1* genes, which respectively funnels amino acids into some desired fusel alcohols such as isobutanol and degrades unpleasant VDKs, *B. bruxellensis* isolates from the Beer population probably serve the production of some desired and complex aromas of specific beers.

### Comparative analysis of the primary and acquired subgenome expression in allotriploid isolates

We then studied the expression variation of the coexisting genomes in the allotriploid populations (Beer, Teq/EtOH, and Wine 1). We specifically focused on the expression of the orthologous genes annotated in both primary and acquired subgenomes in each population, and whose expression was highlighted through a competitive reads mapping strategy (Supplementary Fig. 4a). If all haplotypes would be expressed at the same level, one would expect about one-third of reads mapped on the acquired genome and two-thirds on the diploid primary genome. For each allotriploid population, we observed that the number of reads attributed to the acquired haploid genome significantly deviated and exceeded this expectation (Z-test *P*-value < 2.2e^−16^ for each population), with a median value of 40% of reads mapped to the acquired genome [(38.8–46.0%) for the (10–90%) quantile range] (Supplementary Fig. 4b). This reveals that the haploid subgenome is globally more expressed than each of the two copies of the diploid primary genome. We also observed that this intensified expression is more pronounced within the Wine 1 population, compared to the Beer one (avg. 42.8% for Wine 1 vs avg. 39.8% for Beer, t-test assuming unequal variance *P* = 5.6e^−05^).

**Fig. 5.**
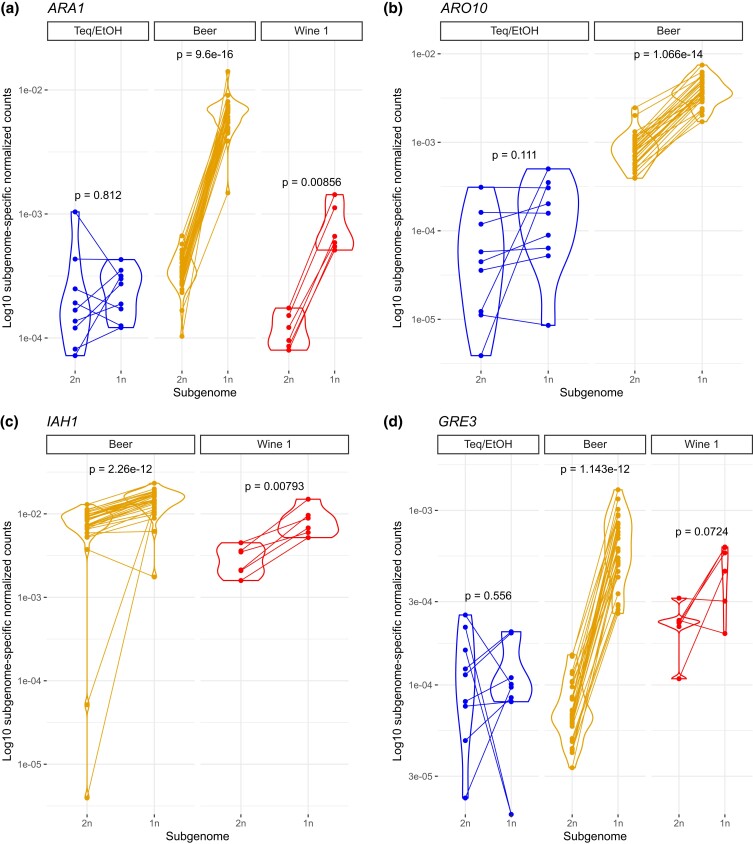
Differential expression between subgenomes within allopolyploid populations. a and b) Expression of *ARA1* and *ARO10* genes in the primary (2n) and acquired (1n) subgenomes of allotriploids. Expression of *ARO10* in Wine 1 is not shown because the considered gene is not present in the haploid subgenome of Wine 1. c and d) Expression of *IAH1* and *GRE3* in the subgenomes of allotriploids. Expression of *IAH1* in Teq/EtOH is not shown because the considered gene is not present in the haploid subgenome of Teq/EtOH. *P*-values are from paired t-tests assuming unequal variance (paired Welch's t-test). Note that in the manuscript, we use the following rationale to consider a gene consistently differentially expressed between subgenomes in a population: it significantly deviates from the 2n versus 1n sample-specific relationship in most samples of the focal population (>7/9 Teq/EtOH samples; >25/30 Beer samples; and >5/6 Wine 1 samples). Provided at least one population showed consistent differential expression between its subgenomes, *P*-values are shown for all three populations, without regarding whether other(s) population(s) fulfilled these per-population criteria of consistent differential expression between subgenomes.

Genes whose expression deviates from the global 2n versus 1n relationship (inferred from sample-wise expression of all 2n and 1n ortholog pairs) were searched for each sample (Supplementary Table 6). Interestingly, the *ARO10* and *ARA1* genes were both over-expressed in the acquired genomes of the Beer isolates compared to the primary genome (Fig. 5a and b). The *GRE3* gene, which is involved in α,β dicarbonyl compounds reduction with methylglyoxal as a substrate, showed the same pattern in this population (Fig. 5d). This compound also contributes to alcoholic beverage aroma, giving an unpleasant taste (Wang and Ho 2012). We noticed that *ARA1* was also over-expressed in the acquired genome of the Wine 1 population. One of the two annotated *IAH1* paralogs (locus_tag=DEBR0S6e08262g) followed the same pattern with haploid subgenomes of both Beer and Wine 1 populations over-expressing it (Fig. 5c). IAH1 encodes an enzyme that hydrolyzes an ester called isoamyl acetate, which displays a pleasant banana aroma but only if present at low concentration (Ferreira and Guido 2018). The second paralog of *IAH1* (locus_tag=DEBR0S6e08240g) displays an overall reduced expression by a median of 76-fold, and this gene was not identified as consistently DE between subgenome in any allotriploid group (Supplementary Fig. 5). Overall, the fact that one copy of the *IAH1*gene is expressed at higher levels in the acquired Beer subgenome, together with similar patterns seen for *ARO10*, *ARA1*, and *GRE3* genes, indicates complex and intertwined regulatory evolution of flavor-impacting processes in the acquired subgenome of *B. bruxellensis* beer isolates.

Finally, regarding genes that deviate from the sample-specific diploid versus haploid expression relationship through lower haploid expression, many of them were found in the Teq/EtOH population (31 among the 62 cases where 1n < 2n expression was consistently observed—Supplementary Table 6). Interestingly, we identified that the *COX7* gene encoding mitochondrial respiratory complex IV subunit and the mitochondrial thioredoxin gene *TRX3* were expressed at a surprisingly low level from the acquired haploid genome in this population. These two genes were also expressed at lower levels by the haploid subgenome of the Beer and Wine 1 clades (Supplementary Fig. 6) compared to their primary genome counterparts, but with a less dramatic effect, consistent with our analysis.

## Discussion

The exploration of gene expression variation within the *B. bruxellensis* species clearly highlighted the impact of the presence of an additional acquired genome on the transcriptional landscape in allopolyploids. Moreover, independent hybridization events gave rise to allotriploid populations characterized by distinct signatures at the transcriptomic level. Finally, these variations are mainly related to the expression of genes located on the acquired genome.

The result of an allopolyploidization event is a fusion of divergent parental genomes into a single nucleus. In most cases, one of the parental subgenomes will retain most of its initial gene content and will be expressed at a higher overall level compared to the other subgenome. By exploring the transcriptional landscape of a significant number of *B. bruxellensis* isolates with different polyploidization trajectories, we revealed higher than expected haploid subgenome expression in each allopolyploid population compared to the primary diploid subgenome. Different mechanisms have been proposed to explain which of the parental genome become dominant after hybridization, mainly from studies performed on plants (reviewed in Bird *et al*. 2018).

These mechanisms include epigenetic modifications (Li *et al*. 2014; Bird *et al*. 2018) and can partly be explained by the density of transposable elements (Cheng *et al*. 2016). It has also been shown that some specific cellular functions are supported by a biased expression of the different parental haplotypes (Timouma *et al*. 2021). It is however difficult to disentangle the role of selection in the specific maintenance of the expression of a parental genome because other processes can lead to similar signatures (Duan *et al*. 2023). Finally, although our study clearly demonstrates that allopolyploidization events led to important transcriptomic changes in *B. bruxellensis*, the larger question of whether or not these changes will ultimately be accommodated by returning to a diploid-like state is still under debate. In this regard, we note that few *B. bruxellensis* aneuploids are found compared to *S. cerevisiae* (Peter et al. 2018; Gounot *et al*. 2020), potentially suggesting to a lower tolerance to genetic imbalance in *B. bruxellensis*. Recognizing that consequences and mechanisms of resolution may be different after aneuploidization versus polyploidization, it is still possible to interpret the strong transcriptomic influence of subgenomes that we observed between allopolyploids groups either as a signature of recent hybridizations, or as a consequence of the expression of an extra haploid genome conferring certain advantages to the allopolyploids.

Beyond the clear evolution of the transcriptional landscape of allopolyploids, we have also identified genes and biological processes that are differentially regulated across populations. Genes involved in transmembrane amino acids transport, such as the allantoate and dipeptides transporter DAL5, are globally upregulated in the Wine 1 and Wine 3 clades. Genes involved in cysteine transport (i.e. the two paralogs of both the *YCT1* and *MUP1* genes) as well as genes involved in nitrogen metabolism and amino acid biosynthesis are also overexpressed in the Wine 3 population, which contrasts sharply with their lower expression in the Beer clade. Nitrogen is known to be in limited amounts in wine must and is mostly found in the form of poor sources such as GABA, leucine, proline, or allantoin (Gutiérrez *et al*. 2015; Becerra-Rodríguez *et al*. 2020). In these conditions, the *DAL5* gene has already been described as overexpressed, as observed in the Wine 3 population (Becerra-Rodríguez *et al*. 2020). The SPS system (Ssy1p-Ptr3p-Ssy5p) in yeast is also known to serve as a sensor for extracellular amino acids, and deficiency in some of these can trigger the expression of specific transporters, such as MUP1, which transports both cysteine and methionine. (Bianchi *et al*. 2019). Unlike wine must, beer wort is generally high in nitrogen (in the form of free amino acids), due to malting and brewing processes, suggesting that these differential expression patterns related to genes involved in amino acid transport and biosynthesis probably reflect an adaptation of *B. bruxellensis* to nitrogen availability in these different ecological niches. As a future direction regarding this putative adaptation to nitrogen availability, it would be interesting to couple transcriptomic and growth-related phenotypes under different culture conditions, as it has been done in *S. cerevisiae* regarding its nitrogen consumption when different amino acid sources are present (Cubillos *et al*. 2017).

In addition to amino acid transport, our study highlighted that mitochondrial genes involved in the electron transport chain and in the redox homeostasis are either globally downregulated in the Teq/EtOH population (as observed for *COA6*) or specifically underexpressed by the corresponding haploid subgenome (as observed for *COX7* and *TRX3*). Both *COX7* and *COA6* are involved in the proper assembly of the mitochondrial complex IV, and their buffered expression in Teq/EtOH samples could impact the oxidative state of the cell by altering electron leakage. In this regard, we might speculate that downregulation of one of the allelic copies of the oxidative stress-related *TRX3* gene, as observed in the Teq/EtOH population, could be a way to cope with such changes.

*Brettanomyces bruxellensis* is used in the production of some craft beers or local special ones such as lambic beers from Belgium (such as Gueuze) (Serra Colomer *et al*. 2019; De Roos and De Vuyst 2019). This more complex composition allows these beers to develop their unique and complex flavor profiles. Among the identified transcriptomic signatures, three genes related to aroma production, namely *ARO10*, *IAH1*, and *ARA1*, are overexpressed in isolates of the Beer population compared to isolates coming from the wine populations. ARO10 and IAH1 are key cytosolic enzymes that catalyze reactions involving fusel alcohols and their derivates, characterized by non-neutral and often strong aromas. ARO10 contributes for example to the production of isobutanol, depicted as bitter, green, harsh but sought after in certain craft beers mentioned above (Piškur and Compagno 2014). Regarding IAH1, it degrades isoamyl acetate, which is an ester that derives from the isoamyl alcohol (Quilter *et al*. 2012; Holt *et al*. 2019; Li *et al*. 2020). Interestingly, the ratio of isoamyl alcohol—which displays a banana flavor—to isoamyl acetate contributes to the final taste of lagers (Ferreira and Guido 2018). Finally, ARA1 contributes to the regulation of the content of undesirable compounds by degrading undesirable VDKs, which are produced at much higher levels from beer compared to wine wort. More importantly, our results also showed that these flavor-contributing genes, such as *ARO10*, *IAH1*, *ARA1*, and also *GRE3*, are all overexpressed in the acquired genome compared to the primary genome of the Beer clade.

Altogether, our study provides insights into the impact of anthropized ecological niches on the expression regulation of the acquired subgenome of allopolyploids. The selection of *B. bruxellensis* is intended to develop specific traits such as the production of complex aromas in the context of beer brewing and may have shaped some of the differences in terms of subgenomes expression we observed. These observations are consistent with previous studies performed on natural or engineered hybrids of relevant yeast species, which show that certain processes are supported by unbalanced expression of the two different parental haplotypes. (Bolat *et al*. 2013; Lairón-Peris *et al*. 2020; Timouma *et al*. 2021; De la Cerda-Garcia Caro *et al*. 2022).

## Supplementary Material

jkad115_Supplementary_Data

## Data Availability

The Illumina reads are available in the Sequence Read Archive under the BioProject PRJEB60461. Raw counts are available as supplementary files and are related to mapping to the primary diploid subgenome (Supplementary File S1), or mapping to the acquired haploid subgenome (Supplementary File S2) or represent the collapse of both counts (Supplementary File S3). Supplemental material available at G3 online.
