# Supplementary material for: Impact of the acquired subgenome on the transcriptional landscape in Brettanomyces bruxellensis allopolyploids
Source: G3 (Bethesda). 2023 May 24;13(7):jkad115. doi: 10.1093/g3journal/jkad115 (PMC10320193; doi:10.1093/g3journal/jkad115)
Supplement: jkad115_Supplementary_Data [file jkad115_supplementary_data.zip › Supplemental_Figures_G3-2023-404281.docx]

**
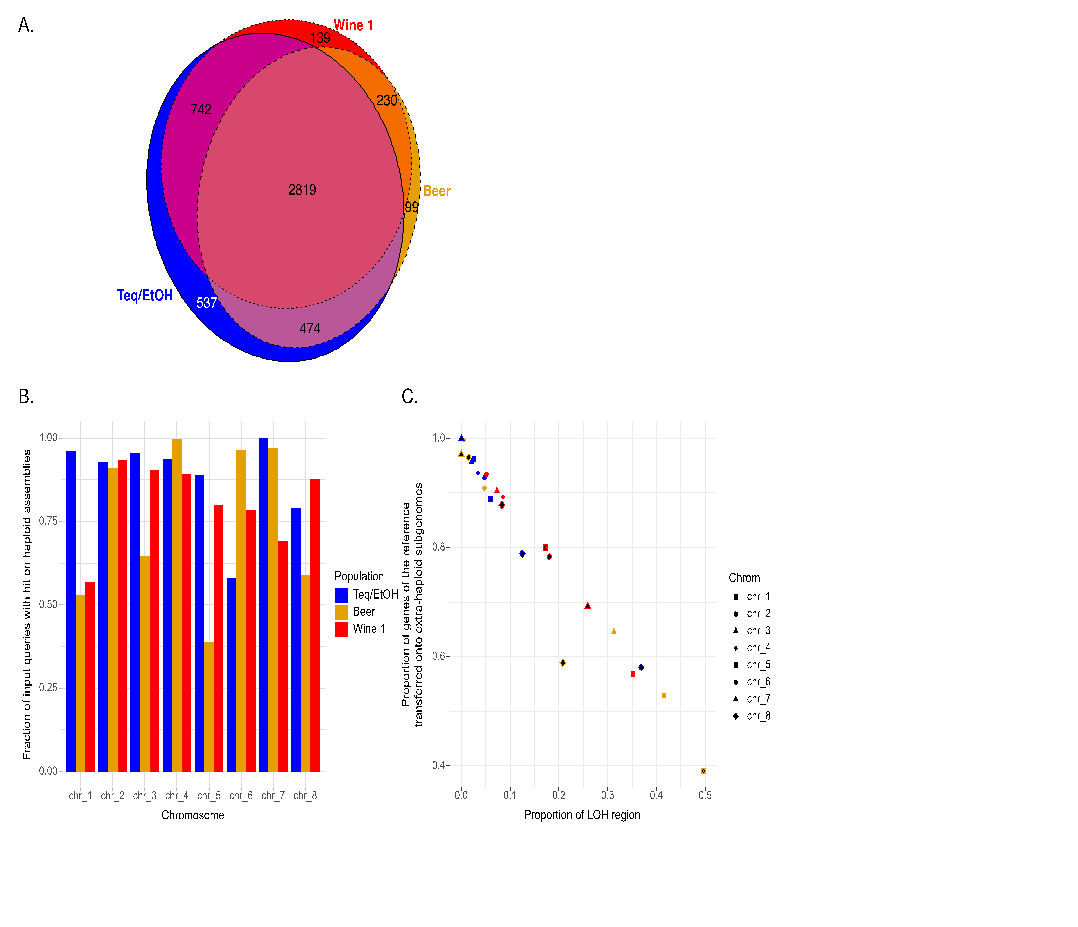
**

**Figure S1. Orthologous gene transfer from the reference diploid genome to the acquired haploid subgenomes. A.**Venn diagram showing the number of protein-coding genes that have been transferred from the *B. bruxellensis* reference genome onto the three acquired haploid (1n) subgenomes (Teq/EtOH, Beer and Wine 1) using BLAST similarity searches. Areas are proportional to the number of transferred genes. **B.** For each chromosome of the reference, the fraction of protein-coding genes that have been transferred onto acquired subgenome assemblies is represented. **C.** Correlation between the fraction of protein-coding genes of the reference transferred onto acquired 1n subgenomes and the extent of loss of heterozygosity (LOH) in these subgenomes (Eberlein et al. 2021). As in panel B. “chromosome” refers to the input gene chromosomal location along the reference genome.

**
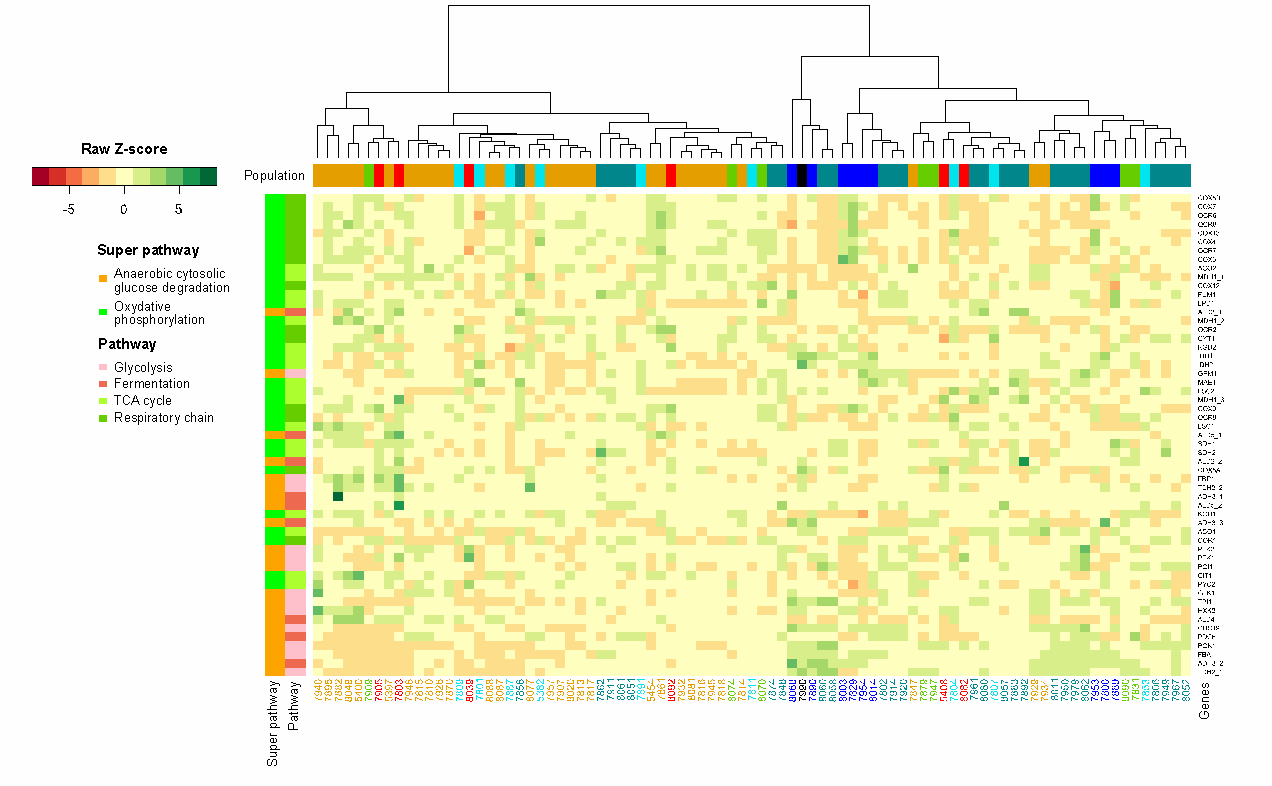
**

**Figure S2. Expression of genes involved in glucose catabolism across populations.** Each row of the heatmap represent a gene, with its expression level normalized by Z-normalization. All 87 samples (columns) are included and columns colors represent populations. On the left, vertical bars highlight the belonging of the genes to the two focal super pathways: “Anaerobic cytosolic glucose degradation” (orange) and “Oxidative Phosphorylation” (green). Each of these two super pathways is refined into two pathways: “Glycolysis” and “Fermentation” for the “Anaerobic cytosolic glucose degradation” super pathway, and “TCA cycle” and “Respiratory chain” for the “Oxidative Phosphorylation” super pathway. Dendrogram at the top was created with the complete link method using Euclidean distance.

**
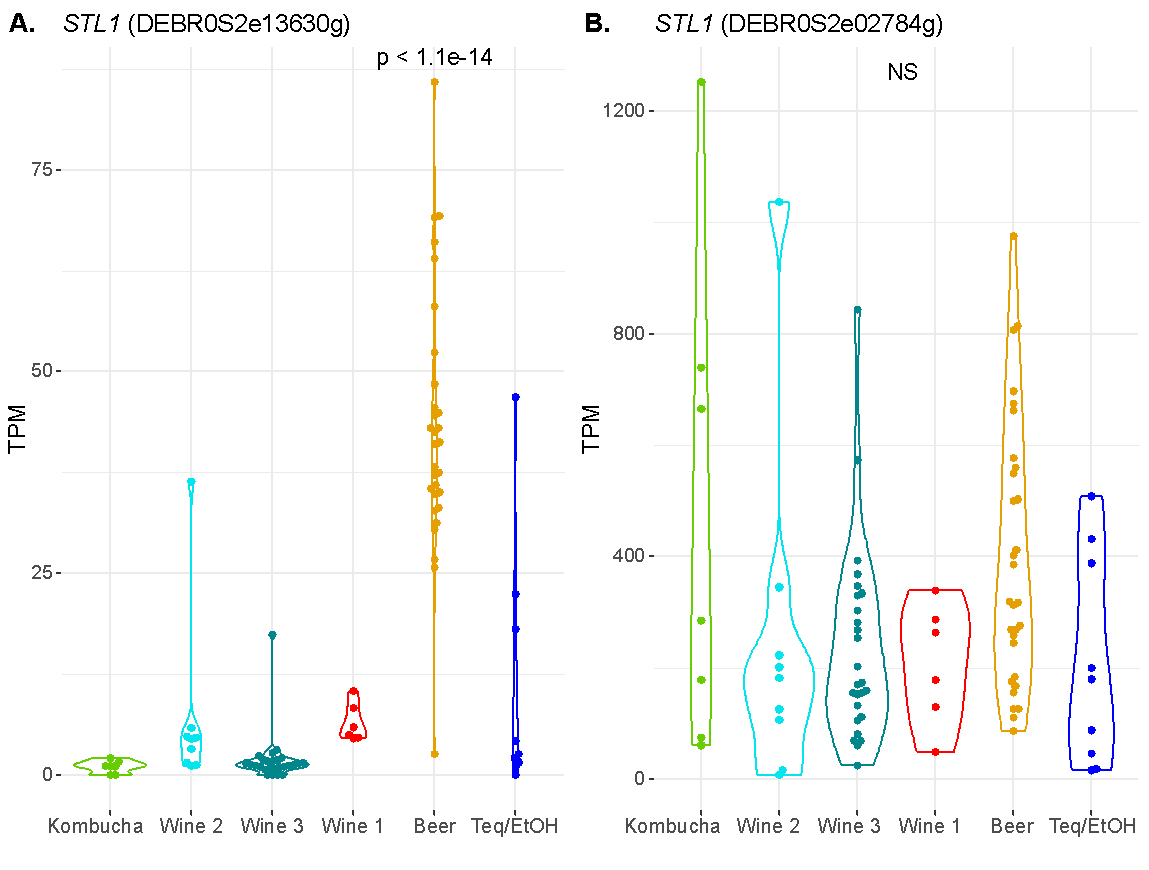
**

**Figure S3. Expression levels of the two *STL1* gene paralogs across populations.** Collapsed expression level is given in TPM. The two paralogs of the glycerol proton symporter *STL1* are expressed at very different levels. **A.** The *STL1* paralog DEBR0S2e13630g is almost not expressed except in the Beer clade where it is consistently expressed at higher expression levels compared to other groups. As mentioned in the manuscript, our analysis revealed that this paralog of *STL1* is a transcriptomic signature of the Beer population. The p-value indicates the significance of testing expression level in the Beer group against other groups as a whole, using a t-test assuming unequal variance (Welch’s t-test). **B.** The other *STL1* paralog (DEBR0S2e02784g) is expressed uniformly at relatively high levels (median expression of 250 TPM), with not any pairwise comparison across populations significant for this paralog.


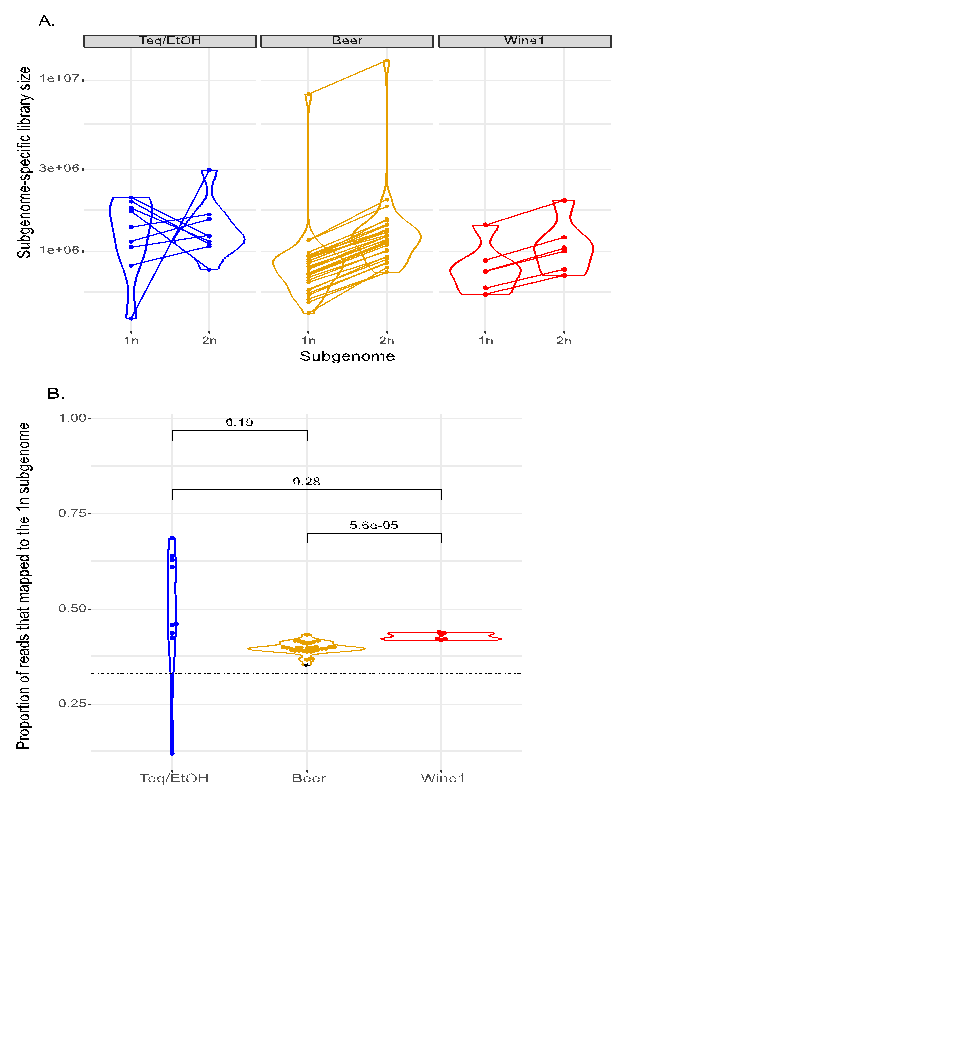


**Figure S4. Relative subgenome library sizes from 2n - 1n orthologous gene sets. A.** Genes with orthologs in both the primary (2n) and the acquired (1n) subgenomes of the considered allotriploid population were used to calculate the number of reads (library size) that were assigned to each subgenome. Lines joining subgenome-specific library sizes corresponds to strains. **B.** Ratio between the sum of reads assigned to the 1n subgenome and the total read counts (*i.e.,* associated to both subgenomes), limited to genes with orthologs in both the primary (2n) and the acquired (1n) subgenomes The dotted line corresponds to the expected ratio of 1/3. Gene sets used are as follows: 4,572 orthologous pairs for the Teq/EtOH group, 3,622 orthologous pairs for the Beer group, and 3,930 orthologous pairs for the Wine 1 group (see Figure S1 above). P-values indicate the FDR-corrected significance of pairwise t-tests of mean ratios across populations (Welch’s t-test assuming unequal variance).


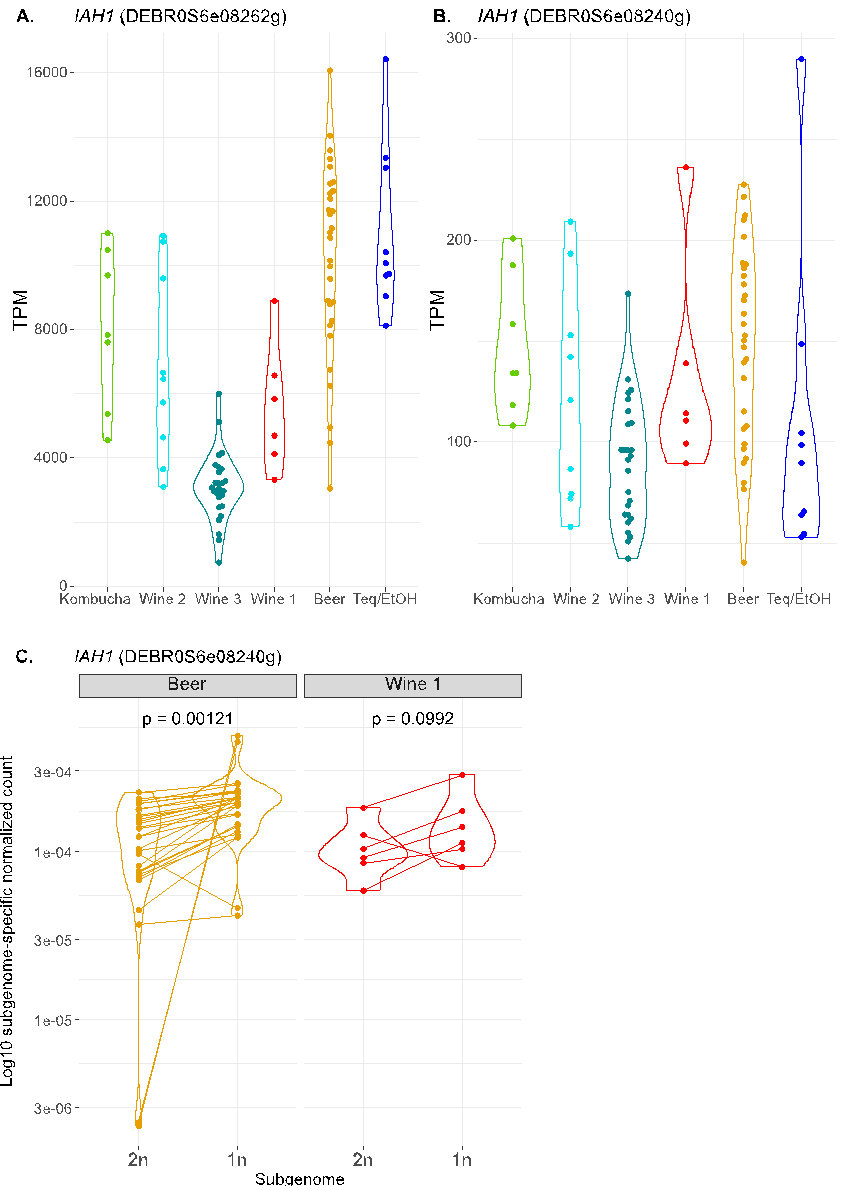


**Figure S5. Expression of the isoamyl-acetate esterase** **(*IAH1*) paralogs across populations and subgenomes. A.** Collapsed expression level of the paralog DEBR0S6e08262 of *IAH1* across populations. **B.** Collapsed expression level of the paralog DEBR0S6e08240 of *IAH1* across populations. Expression levels of the two paralogs are given in TPM and span very different ranges of expression (TPM _IAH1_DEBR0S6e08262_ >> TPM _IAH1_DEBR0S6e08240_). **C.** The *IAH1*_DEBR0S6e08240 paralog does not reach the threshold of at least 25 out the 30 Beer samples expressing it with a significant deviation from the sample-specific 2n versus 1n relationship, contrary to *IAH1*_DEBR0S6e08262 (see Figure 4A). Only two clearly visible samples meet this criterion (shown p-values are from paired Welch’s t-test, run without regarding if our per population criteria was met). Expression values in the Teq/EtOH population are not shown because *IAH1*_DEBR0S6e08240 is not present in the Teq/EtOH haploid subgenome.

**
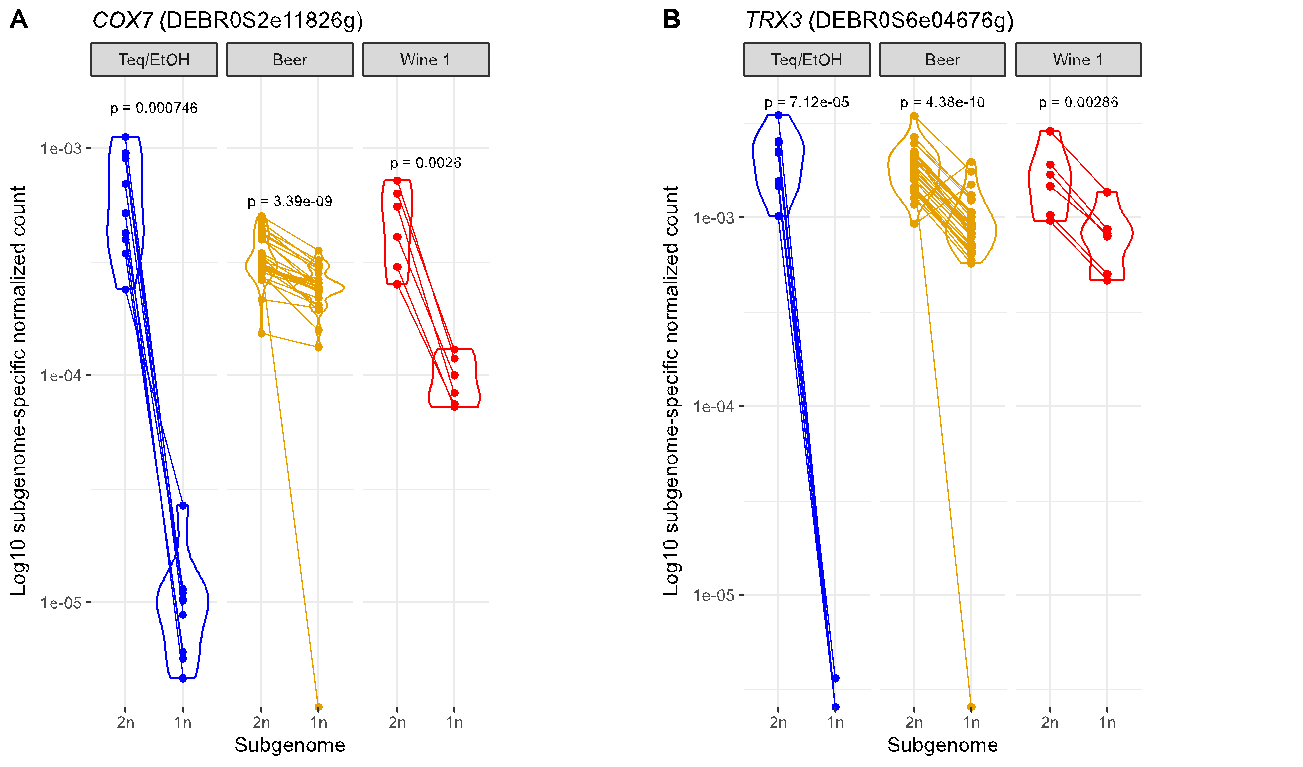
**

**Figure S6. Differential expression between subgenomes through haploid expression buffering. A.** Expression of *COX7* gene (normalized by subgenome-specific library size) in diploid (2n) and haploid subgenomes (1n) of allopolyploids, grouped by population. **B.** Expression of *TRX3* gene (normalized by subgenome-specific library size) in subgenomes of allopolyploids, grouped by population. For all panels, p-values are from paired t-tests assuming unequal variance (paired Welch’s t-test). Note that in the manuscript, we use the following rationale to consider a gene consistently differentially expressed between subgenomes in a subpopulation: it has to significantly deviate from the 2n versus 1n sample-specific relationship in most samples of the subpopulation (> 7/9 Teq/EtOH samples; >25/30 beer samples; > 5/6 wine 1 samples). For both *COX7* and *TRX3*, and despite the significant pair Welch’s t-test p-values in all allopolyploid populations, these criteria were only met for the Teq/EtOH population.
